# Role of Basal Ganglia Circuits in Resisting Interference by Distracters: A swLORETA Study

**DOI:** 10.1371/journal.pone.0034239

**Published:** 2012-03-28

**Authors:** Perrine Bocquillon, Jean-Louis Bourriez, Ernesto Palmero-Soler, Alain Destée, Luc Defebvre, Philippe Derambure, Kathy Dujardin

**Affiliations:** 1 University of Lille Nord de France, Lille, France; 2 Lab. Neurosciences Fonctionnelles et Pathologies, Lille, France; 3 Clinical Neurophysiology Department, Lille University Medical Center, Lille, France; 4 Eemagine Medical Imaging Solutions GmbH, Berlin, Germany; 5 Neurology and Movement Disorders Department, Lille University Medical Center, Lille, France; Hangzhou Normal University, China

## Abstract

**Background:**

The selection of task-relevant information requires both the focalization of attention on the task and resistance to interference from irrelevant stimuli. Both mechanisms rely on a dorsal frontoparietal network, while focalization additionally involves a ventral frontoparietal network. The role of subcortical structures in attention is less clear, despite the fact that the striatum interacts significantly with the frontal cortex via frontostriatal loops. One means of investigating the basal ganglia's contributions to attention is to examine the features of P300 components (i.e. amplitude, latency, and generators) in patients with basal ganglia damage (such as in Parkinson's disease (PD), in which attention is often impaired). Three-stimulus oddball paradigms can be used to study distracter-elicited and target-elicited P300 subcomponents.

**Methodology/Principal Findings:**

In order to compare distracter- and target-elicited P300 components, high-density (128-channel) electroencephalograms were recorded during a three-stimulus visual oddball paradigm in 15 patients with early PD and 15 matched healthy controls. For each subject, the P300 sources were localized using standardized weighted low-resolution electromagnetic tomography (swLORETA). Comparative analyses (one-sample and two-sample t-tests) were performed using SPM5® software. The swLORETA analyses showed that PD patients displayed fewer dorsolateral prefrontal (DLPF) distracter-P300 generators but no significant differences in target-elicited P300 sources; this suggests dysfunction of the DLPF cortex when the executive frontostriatal loop is disrupted by basal ganglia damage.

**Conclusions/Significance:**

Our results suggest that the cortical attention frontoparietal networks (mainly the dorsal one) are modulated by the basal ganglia. Disruption of this network in PD impairs resistance to distracters, which results in attention disorders.

## Introduction

Attention underlies most cognitive processes and is therefore a key issue in neuropsychology. Attention can be focused by relevant signals derived from task demands (target stimuli) or captured by salient properties of stimuli that are sometimes irrelevant for the task (distracter stimuli) ([1], [2]. During a task, input selection allows the preferential processing of some sources of information from the internal or external environment at the expense of other stimuli [3]. According to Luck and Gold (2008), input selection can further be divided into the control of selection (i.e. the determination of which inputs have to be selected) and the implementation of selection (i.e. the process of enhancing the target inputs and suppressing distracter inputs).

Previous studies have shown that distracter and target processing activities are subserved by different (but probably interconnected) functional circuits. Indeed, two specific networks have been identified: (i) a dorsal frontoparietal (DFP) network connecting the dorsal parietal cortex to the dorsal frontal cortex, (ii) a ventral frontoparietal (VFP) network connecting the temporoparietal junction to the ventral prefrontal cortex [4]. The DFP network may generate and maintain endogenous signals on the basis of current goals [4]. This network is involved in processing targets and distracters but is most prominent during the latter activity [5], . According to Bledowski et al. [5], this network may reflect a disengagement of attention previously focused on the target detection and the subsequent allocation of attentional resources to the salient distracter stimulus. The VFP network is not activated by expectation or task preparation but is reportedly engaged in the detection of rare events [5]–[7]. Hence, the VFP network responds (along with the dorsal network) when behaviorally relevant objects or targets are detected [4], [7], [8]. This has already been shown for visuospatial attention [8]. The VFP is indeed activated when attention is reoriented to a target occurring at an unexpected location (an invalid target). However, its involvement in non-spatial attention has also been evidenced when a target appears infrequently, as in an oddball paradigm [4], [5], [6].

Most of these data come from neuroimaging studies (essentially fMRI) but other methods may be of use for investigating the involvement of cortical networks in attention processes. This is particularly true for distributed source localization of cognitive event-related potentials (ERPs) in general and P300 in particular [6], [9]. The P300 potential is a positive component occurring around 300 ms after a stimulus. It is usually considered to reflect attention and working memory [10] and occurs when a subject has to detect an awaited and unexpected stimulus. This is the case in an “oddball” paradigm in which low-probability target and non-target stimuli are mixed with high-probability standard stimuli [11]–[13]. The P300's amplitude corresponds to the attentional resources allocated to the task, whereas the potential's latency is thought to correspond to the time needed to evaluate a stimulus' characteristics. In three-stimulus oddball paradigms with standard, infrequent non-targets (also referred to as distracters) and infrequent target task-relevant stimuli (i.e. targets), two main P300 components can be identified: an early, frontocentral component (often called P3a) that occurs when the subject is presented with a distracter stimulus and a later, centroparietal component (often called P3b) that follows presentation of a target stimulus [13]–[17]. The early, frontal component is thought to correspond to evaluation of the stimulus or an attention alert [18], [19]. It is further thought to correlate with the orienting response and reflects an involuntary switch of attention (i.e. attention reallocation) from the main task (target/standard categorization) to a deviant, non-target stimulus [7], [20]–[22]. This attention reallocation could also be induced directly by deviance of the distracter from the stimulus context, without the need for cognitive interference with the ongoing task [23]. In this hypothesis, the distracter P3a would thus reflect the neural response of attentional capture. However, other researchers believe that the P3a also reflects inhibition of an automatic response to the salient but task-irrelevant stimulus [10], [22]. The later, more posterior component is variously thought to be related to (i) restructuring of working memory following the presentation of new information [24], [25], (ii) the decision on how to respond [13], [26] and (iii) the stimuli's access to global-workspace processing [27]. The P3b component reflects attentional processes that enable information to be categorized [11], [24], [25]. Experiments in healthy subjects (either with fMRI or with standardized-weighted low resolution electromagnetic tomography (swLORETA)) suggest that the distracter- and target-elicited P300 components have different brain sources [5], [6]. Indeed, both approaches have shown that the distracter-elicited ERP mainly involves sources in the DFP network, whereas target-elicited ERPs also recruit the VFP network.

The aforementioned data suggest significant cortical involvement in attentional processes. However, the contribution of subcortical structures in these cognitive functions is still unclear. The cortical areas involved in these frontoparietal attentional networks are strongly linked to the striatum via several basothalamocortical circuits [28]–[30]. The head of the caudate, the dorsolateral prefrontal (DLPF) cortex and posterior parietal cortex form an executive loop, whereas the ventrolateral prefrontal (VLPF) cortex, temporal cortex and the body and tail of the caudate nucleus are involved in a visual loop. It is thus very likely that striatal disruption will disorganize the attentional frontoparietal networks identified by Corbetta [4], [8] and therefore result in attention disorders.

Basal ganglia damage (such as seen in Parkinson's disease (PD)) may represent a novel setting for investigating this question. Parkinson's disease is a neurodegenerative condition characterized by loss of dopaminergic cells in the substantia nigra pars compacta. It causes hallmark motor symptoms associating bradykinesia, rest tremor and rigidity [31]. Patients also develop non-motor symptoms, including cognitive impairment. In particular, attention disorders are typically seen in PD - even in the early stages of the disease. Nevertheless, it is still not clear whether this impairment results from lower recruitment of attentional resources or defective inhibition of irrelevant events [32]–[35]. The P300 potential has already been used to study attention impairment in PD. Most of these previous studies evidenced a reduced P300 amplitude [34], [36]–[42] and a longer P300 latency [36], [41], [43]–[52]. In contrast, other similar work did not evidence these changes [43], [53]–[57]. However, most of these studies involved conventional two-stimulus oddball paradigms, which prevented the separate identification of distracter and target-elicited P300 components. To the best of our knowledge, source analysis of P300 components and functional imaging have never previously been performed in PD patients performing oddball paradigms. However, high-resolution (128-channel) EEG recording for source analysis provides an excellent time resolution (higher than fMRI), combined with a good spatial resolution (higher than standard EEG). This allows accurate detection of anatomical substrates for short-time cognitive processes as in oddball paradigms.

The aim of the present study was to investigate how the basal ganglia might modulate the functioning of the DFP and VFP attentional networks during distracter and target processing.

## Methods

### Objectives

The objective of this study was to determine the nature of attentional impairment in early PD by combining the investigation of the P300 components' usual features (amplitude and latency) with the identification of their cortical generators in a swLORETA source analysis [58], [59]. If the attentional impairment in PD results from deficient implementation of selection, this would lead to difficulty in resisting interference from distracter stimuli and would thus change the characteristics of the distracter-elicited P300 (namely its swLORETA generators). Alternatively, if attention impairment in PD is due to an alteration in control of selection, this would result in the failure of target detection and would prompt changes in the characteristics of the target-elicited P300 (again, its swLORETA generators).

### Participants

The study included 15 right-handed patients (ten males and five females) with probable PD according to international criteria [60]. All patients were treated and assessed after receiving their usual anti-parkinsonian medication (eight received dopaminergic agonists only, two L-Dopa only and five had a treatment associating dopaminergic agonists and L-Dopa). The mean L-Dopa equivalent daily dosage [61] is shown in Table 1. Patients with motor fluctuations, a tremor subscore (item 20) above 2 on the UPDRS III scale, undergoing deep brain stimulation or suffering from depression or dementia (according to the DSM IV-TR [62] and PD dementia criteria [63], respectively) were excluded from the study. Fifteen right-handed healthy controls (HCs) (eight males and seven females) also participated. According to self-reports, the subjects had no history of psychiatric problems and were not taking any psycho-active drugs. The HCs were also free of neurological disease. The two groups were matched in terms of age, gender and duration of formal education.

**Table 1:** Clinical and demographic features of the Parkinson's disease (PD) patients and healthy controls: mean (standard deviation).

	PD patients	Healthy controls	p
Age (years)	59.2 (6.4)	59.1 (7.4)	0.979
Gender ratio (M/F)	10/5	8/7	0.456
Duration of education (years)	12.5 (2.4)	12.7 (3.2)	0.966
Mattis Dementia Rating Scale (out of 144)	141.3 (2.7)	142.1 (1.6)	0.642
MADRS score	3.1 (2.2)	2.2 (4.5)	0.029
Hoehn and Yahr score	1.5 (0.5)		
UPDRS III score	18.6 (8.7)		
Mean (SD) L-Dopa equivalent daily dose (mg/d)	542 (222)		
Time since disease onset (years)	4.8 (3.5)		

p values were determined in t-tests (except for the gender ratio, for which a χ^2^ test was applied).

Table 1 summarizes the subjects' demographic and clinical features.

All participants were free of visual impairments, according to the Early Treatment Diabetic Retinopathy Study scale (ETDRS research group, 1991). An extensive cognitive examination, including an assessment of the overall cognitive status (Mattis Dementia Rating Scale [64] and the main cognitive domains (detailed in the Supporting Information S1) enabled us to rule out cognitive decline or dementia. The severity of anxiodepressive symptoms was assessed on the Montgomery and Asberg Depression Rating Scale (MADRS) [65].

### Description of procedures

#### Task and recording procedure

Subjects were comfortably seated and watched a 17-inch monitor set 150 cm away. Event-related potentials were recorded as the subjects performed a 3-stimulus visual oddball paradigm similar to that used by Bledowski et al. [66].

A session included two different task types (a circle task with squares as distracters and a square task with circles as distracters) with 360 stimuli each. The order of the two task types was counterbalanced so that half the participants saw circles first and half saw squares first.

**Figure 1:**
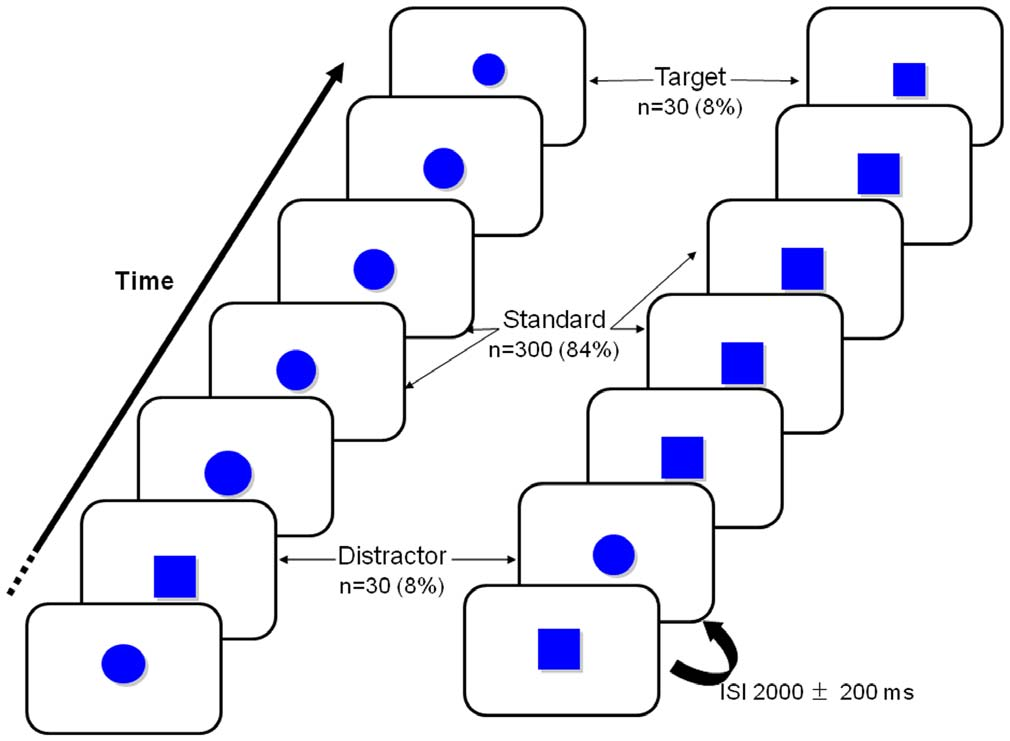
A schematic representation of the three-stimulus visual oddball paradigm (with the circle task on the left and the square task on the right).

Figure 1 depicts the experimental task: the stimuli were solid blue shapes displayed in a semi-random order for 75 ms each. The interstimulus interval varied from 1800 to 2200 ms. The stimuli were defined as standard shapes (40 mm diameter circles or 35 mm sided squares), distracters (a different shape: 35 mm sided squares or 40 mm diameter circles, respectively) or targets (smaller than the standard shape: 33 mm diameter circles or 30 mm sided squares) and were displayed with a probability of 0.84, 0.08, and 0.08, respectively. The subject was told to respond to the target stimuli by pressing a button with his/her right hand within 2000 ms.

This three-stimulus oddball paradigm involves two different attention processes: (i) the size-based detection of a target among standard stimuli in a complex discrimination task, (ii) the shape-based detection of expected vs. unexpected, infrequent stimuli, together with the need to resist interference produced by infrequent distracter stimuli (since the subjects were not told about these distracters in advance).

Before each task, all subjects had a practice run in the absence of distracter stimuli. The mean reaction time, the omission rate, the overall commission rate and the distracter commission rate were recorded. The omission rate was defined as the number of misses divided by the total number of targets (i.e. 60)×100. The overall commission rate was defined as the number of false alarms divided by the total number of non-target stimuli (distracter+standard stimuli, i.e. 660)×100. The distracter commission rate corresponded to the number of false alarms divided by the total numbers of distracters (i.e. 60)×100.

The electroencephalogram (EEG) was recorded from 128 scalp sites using a DC amplifier (ANT Software BV, Enschede, the Netherlands) and a Quick-cap® 128 Ag/AgCl electrode cap (ANT Software BV) placed according to the 10/05 international system, with a linked mastoid reference [67]. The impedance was kept below 5 kΩ. An electro-oculogram (EOG) was recorded to detect artifacts related to eye movements and blinks. The EEG and EOG were digitized with a sampling rate of 512 Hz and recorded with EEProbe® software (ANT Software BV).

#### EEG analysis

The EEG was analyzed with EEProbe® software. The raw data waveforms were band-pass filtered by convolving them with a finite-impulse response filter and a Hamming window. The half-power cut-offs were 0.1 and 30 Hz. EEG epochs that contained eye movements or blink artifacts were automatically detected, then manually classified as either blinks or eye movements and separately corrected with the EEProbe® regression algorithm. Whenever the subject missed a target stimulus or responded to a distracter stimulus, the event was excluded from the EEG analysis. The waveforms (analyzed from 100 ms pre-stimulus to 900 ms post-stimulus) were averaged separately for the standard, distracter and target conditions. In the present study, we chose to refer to distracter- and target-elicited P300 components, rather than the conventional definitions of P3a and P3b. Indeed, distracters and targets can generate P3a and P3b, although P3a components predominate for distracters and P3b predominate for targets [14]–[17]. For each epoch, a baseline correction was performed by using data from 100 ms prior to the stimulus. The P300 components were visually defined as the largest positive deflection in the distracter and target stimulus waveforms within the 250 to 600 ms time window in the 128-channel overlay plot. Detection of the P300 peak was confirmed by calculating the global field power, which represents the signal's power when all electrodes are taken into account [68], [69].

The P300 amplitude was defined as the voltage difference between the baseline and the largest positive wave peak in the analyzed time window. Latency was defined as the time between stimulus onset and the P300 peak. Amplitude and latency measurements were performed for the three midline electrodes (Fz, Cz and Pz).

#### swLORETA P300 source localization

**Figure 2:**
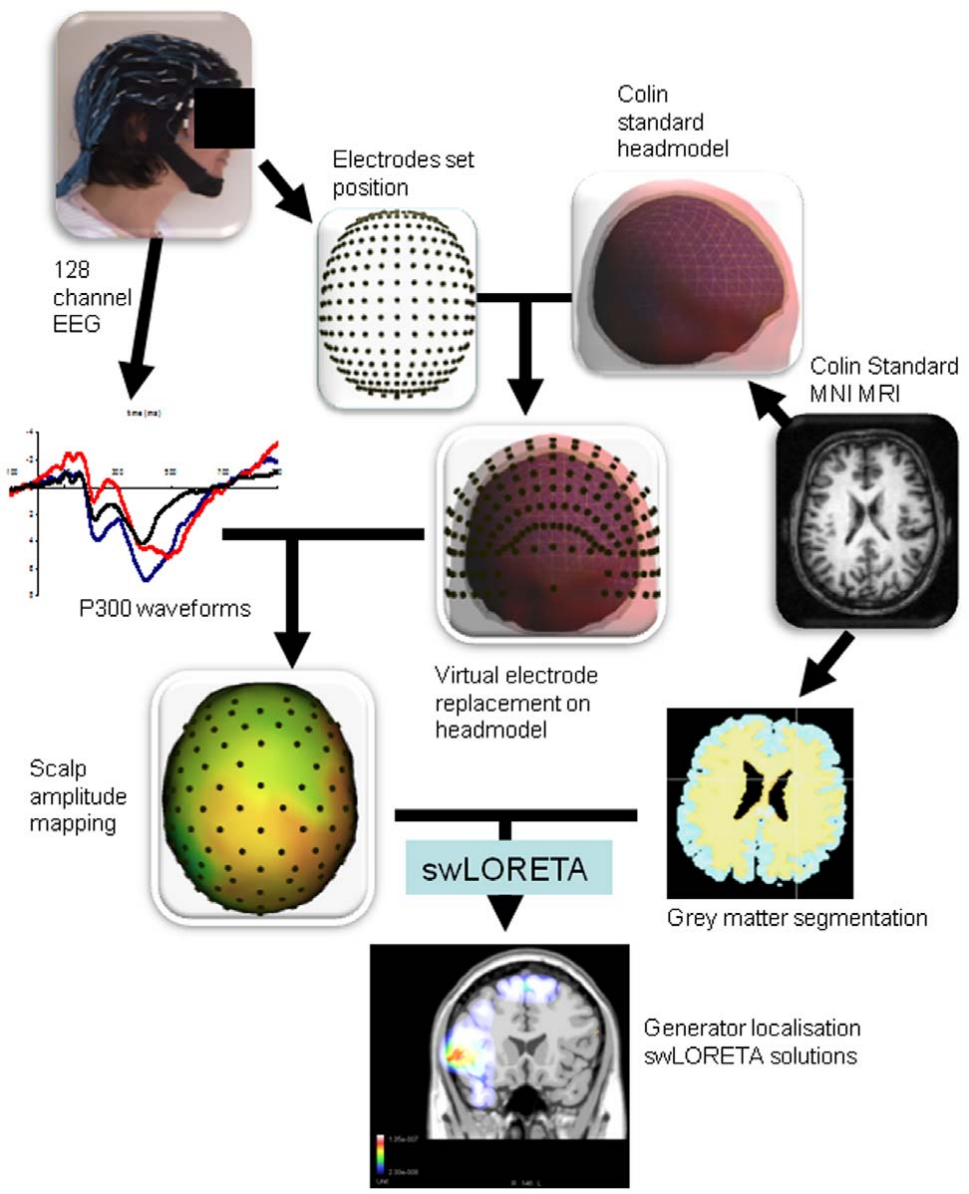
Procedure for the swLORETA P300 source analysis.

In order to study the effect of processing distracter and target stimuli, difference waveforms were calculated by subtracting the standard stimulus waveform from the distracter waveform (to yield the D-S waveform) and target waveforms (to yield the T-S waveform) for each subject and for all scalp-EEG channels. P300 source analysis was performed according to the swLORETA method with ASA® software (ANT Software BV), as shown in Figure 2. swLORETA is a recent update of the standardized low-resolution brain electromagnetic tomography (sLORETA) method introduced by Pascual-Marqui in 2002. This method is a distributed technique that enables the so-called “electromagnetic inverse problem” to be solved. sLORETA is a useful tool for modeling spatially distinct source activities in the absence of prior knowledge of the generators' anatomical location. The sLORETA method generates statistical parametric maps that reflect the reliability of the estimated current source density distribution. It shows exact topographic properties, with a zero-localization error for single dipoles in noiseless simulated data (for more specific details of the method and its experimental validation, see [58], [70], [71]). swLORETA additionally incorporates a singular value decomposition-based lead field weighting that compensates for the sensors' differing sensitivity to current sources at different depths [59]. This weighting enables accurate reconstruction of surface and deep current sources in simulated data - even in the presence of noise and when two dipoles are simultaneously active.

The swLORETA solution was computed using a three-dimensional grid of points (or voxels) representing the possible sources of the signal. Furthermore, the solution was restricted to the grey matter by selecting only voxels in which the grey matter probability was not equal to zero (based on the probabilistic brain tissue maps available from the Montreal Neurological Institute [72]–[74]. Lastly, the 1056 grid points (with a 5 mm grid spacing) and the recording array (128 electrodes) were registered against the Collins 27 MRI map (with a 1 mm spatial resolution) [73]. The Boundary Model was used to compute the lead field matrix. The lead field matrix models the mechanism by which the original current sources are superimposed on each other to produce the measured voltage fields at each detector; this is the first step needed to compute any inverse solution [75].

We calculated the mean value of the swLORETA analysis performed for all time-points within a 40 ms time window around the P300 peak in each difference waveform. The P300 peak was defined as the distracter-elicited P300 latency for D-S waveforms and the target-elicited P300 latency for T-S waveforms.

### Ethics

All study subjects provided their written, informed consent to participation and the study had been approved by the local independent ethics committee (“Comité de Protection des Personnes Nord-Ouest IV”, 2007-A 00227-46,).

### Statistical analysis

#### Behavioral data

Due to a floor effect and the skewness of the distributions, Mann-Whitney tests were used to compare reaction times, omission rates and overall and distracter commission rates in PD patients and HCs. The significance threshold was set to p<0.05.

#### Amplitude data

A three-factor, repeated-measures ANOVA was performed on the P300 amplitude data with group (PD patients or HCs) as a between-group factor and stimulus type (distracter or target) and location as within-group factors. The location factor had nine levels: Fz, Cz, Pz, left and right frontal areas (consisting of channels AFF1, AFF5h, F1, F3, F5, FFC1h, FFC3h, FFC5h, FC1, FC3 and FC5 on the left and AFF2, AFF6h, F2, F4, F6, FFC2h, FFC4h, FFC6h, FC2, FC4 and FC6 on the right), left and right central areas (consisting of channels FCC1h, FCC3h, FCC5h, C1, C3, C5, CCP1h, CCP3h and CCP5h on the left and FCC2h, FCC4h, FCC6h, C2, C4, C6, CCP2h, CCP4h and CCP6h on the right), left and right parietal areas (consisting of CP1, CP3, CP5, CPP1h, CPP3h, CPP5h, P1, P3, P5, PPO1 and PPO5h on the left and CP2, CP4, CP6, CPP2h, CPP4h, CPP6h, P2, P4, P6, PPO2 and PPO6h on the right). A Greenhouse-Geisser correction was applied when the assumption of sphericity was violated. When required, post-hoc analyses with t-tests were performed. The threshold for statistical significance was set to p<0.05.

#### Latency data

A three-factor repeated-measures analysis of variance (ANOVAs) with stimulus type (distracter or target) and location (Fz, Cz and Pz) as within-group factors and group (PD patients or HCs) as a between-group factor was performed on the P300 latency data. A Greenhouse-Geisser correction was applied when the assumption of sphericity was violated. When required, post-hoc analyses with parametric tests were performed. The significance threshold was set to p<0.05.

#### P300 source localization data

All statistical analyses were performed with SPM5® software (Wellcome Department of Cognitive Neurology, Institute of Neurology, University College London, London, UK).

To locate the P300 sources, a rigorous method was used to establish a threshold value for deciding on the statistical significance of the current density. To this end, a one-sample *t*-test was performed for each voxel in the source space; the null hypothesis was that there was no relationship between the mean current density and the changes in our experimental conditions. The null hypothesis can be stated formally as:
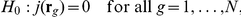
where 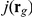 is the modulus of the swLORETA at position 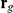 and *N* is the total number of voxels in the source space.

Due to the properties of linear inverse solutions like swLORETA, this null hypothesis will never be satisfied by any voxel; all of the latter will present some differences from zero activity, due to the linear mixing present in the linear inverse solution. This is why the modulus of the swLORETA has to be normalized, in order to distinguish between regional activity (i.e. an intensity change due to a specific stimulation) and overall activity (i.e. an intensity change due to a task unrelated to the stimulation). For each subject, normalization was performed by dividing the value at each voxel by the mean over all voxels and then subtracting a value of one:
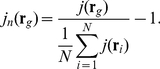
(1)


The mean over all voxels represents the global activity. With this change, our null hypothesis of non-significant activation related to changes in our experimental conditions translates into:
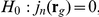


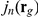
 was defined in Eq. (1). This is similar to the overall normalization used in positron emission tomography (PET) experiments and which is available in the SPM® statistics package [76], [77].

Gaussian smoothing with an 8 mm full-width-at-half-maximum kernel was performed on the normalized current density maps, in order to increase the signal-to-noise ratio and ensure that between-subject differences were assessed on a reasonable spatial scale with regard to the functional anatomy.

One sample t-tests were then applied to D-S and T-S normalized and smoothed swLORETA maps for both groups, with a grey-matter mask; this enabled us to define the sources of the P300 component elicited by distracter and target stimuli, respectively.

Two sample t-tests were used to compare normalized swLORETA maps in patients and controls. This was done with a PD patient-HC contrast (in order to identify P300 generators found in PD patients but not in HCs) and with a HC-PD patient contrast (in order to highlight P300 sources only displayed by HCs).

This operation was performed first for distracter and target conditions as a pooled dataset and then for each condition separately.

In order to avoid false positives in these tests (while not compromising our ability to evaluate functional networks), we applied 125-voxel spatial clustering (corresponding to the spatial resolution (5 mm×5 mm×5 mm) of our source grid) and a low p-value (0.0005 for one sample t-tests and 0.005 for two-sample t-tests. Combining a spatial extent threshold with low p values decreases random activation [78].

The anatomical labels of the source locations were specified in SPM® using a further development of a three-dimensional maximum probability atlas [79], in order to avoid multiple anatomical transformations that would otherwise have led to localization errors.

## Results

### Behavioral results

The median and ranges of behavioral results are shown in Table 2. Mann-Whitney tests revealed a significantly higher distracter commission rate (Z = −2.374, p = 0.018) in PD patients than in HCs. The two groups did not differ significantly in terms of the mean reaction time, omission rate or overall commission rate.

**Table 2:** Behavioral performance of Parkinson's disease (PD) patients and healthy controls: median (range).

	PD patients	Healthy controls	p
Reaction time (ms)	556 (435–757)	550 (439–841)	0.756
Omission rate (%)	10 (0–28)	6.7 (0–23)	0.574
Commission rate (%)	6 (0–38.9)	1.8 (0.6–9.1)	0.329
Commission rate for distracters (%)	0 (0–16.6)	0 (0–1.77)	0.018*

* p<0.05.

### P300 amplitude

Table 3 shows the mean P300 amplitudes in Fz, Cz and Pz for distracter and target stimuli.

**Table 3:** Distracter and target-elicited P300 amplitudes and latencies: mean (standard deviation).

			Distracter			Target	
		Fz	Cz	Pz	Fz	Cz	Pz
Amplitude	PD	5.73 (3.25)	5.96 (3.39)	5.38 (2.84)	3.6 (2.9)	3.8 (2.3)	4.52 (2.04)
(µV)	controls	6.47 (3)	5.58 (2.20)	4.1 (1.81)	5.16 (2.31)	4.77 (2.43)	4.23 (1.87)
Latency	PD	397 (55)	397 (55)	397 (57)	486 (50)	487 (50)	488 (48)
(ms)	controls	362 (58)	362 (58)	362 (60)	427 (53)	423 (49)	423 (48)

**Figure 3:**
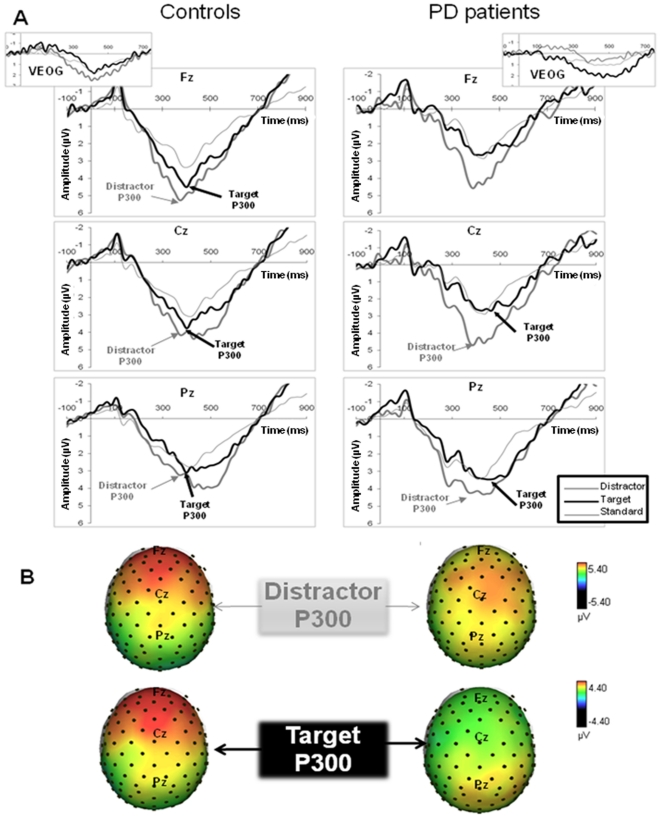
Grand average ERP waveforms and scalp amplitude maps at P300 peak. Top panel (3A): grand average ERP waveforms from three midline electrodes (Fz, Cz, Pz) for standard stimuli (the thin grey line), target stimuli (the thick black line) and distracter stimuli (the grey line), with identification of distracter- and target-elicited P300 components. Data from controls and PD patients are shown on the left and the right, respectively. Bottom panel (3B): scalp amplitude maps at P300 peak latencies for the distracter (top) and target (bottom) stimuli. Data from healthy controls and PD patients are shown on the left and the right, respectively. Dots indicate electrode positions on the scalp.

Figure 3 shows grand averages of ERP waveforms for each stimulus type in Fz, Cz and Pz, with identification of the distracter- and target-elicited P300 components.

The ANOVA revealed a significant main effect of “stimulus type” (F_(1,28)_ = 4.931, p = 0.035), with a larger amplitude for the distracter-elicited P300 than for the target-elicited P300. The analysis also showed a main effect of “location” (F_(8,224)_ = 6.658, p<0.001) and significant “location”×“stimulus type” (F_(8,224)_ = 3.106, p = 0.036) and “location”×“group” (F_(8,224)_ = 4.910, p = 0.002) interactions. No main effect of group was observed. Further analyses of the “location”×“stimulus type” interaction revealed that the distracter-elicited P300 was larger than the target-elicited P300 in the left frontal and central areas (t_29_ = 2.133, p = 0.042 and t_29_ = 2.401, p = 0.023, respectively) and at Fz and Cz (t_29_ = 2.788, p = 0.009 and t_29_ = 2.969, p = 0.006, respectively). The stimulus type did not have a significant effect at the other locations. Regarding the “location”×“group” interaction, further analyses revealed a significant location effect in the HCs (F_(8,112)_ = 8.049, p<0.001) but not in the PD patients (F_(8,112)_ = 2.517, p = 0.087). In HCs, P300 amplitudes showed a frontoparietal gradient. The P300 amplitude was larger at Fz than at Cz (t_14_ = 2.641, p = 0.019) and Pz (t_14_ = 4.216, p = 0.001) and larger at Cz than at Pz (t_14_ = 2.842, p = 0.013). It was larger in left frontal area than in left central and parietal areas (t_14_ = 2.620, p = 0.02 and t_14_ = 3.598, p = 0.003, respectively), larger in the left central area than in the left parietal area (t_14_ = 3.393, p = 0.004), larger in the right frontal area than in the right central and parietal areas (t_14_ = 2.492, p = 0.026 and t_14_ = 3.248, p = 0.006, respectively) and larger in the right central area than in the right parietal area (t_14_ = 2.716, p = 0.017). A median-to-lateral gradient was also seen in frontal and central areas, with a larger amplitude at Fz than in the left and right frontal areas (t_14_ = 2.513, p = 0.025 and t_14_ = 2.184, p = 0.046, respectively) and a larger amplitude at Cz than in left central areas (t_14_ = 2.792, p = 0.014). The other comparisons were not statistically significant.

### P300 latency

Mean latencies at each electrode are displayed in Table 3. ANOVAs revealed a significant main effect of “stimulus type” (F_(1,28)_ = 117.77, p<0.001) and “group” (F_(1,28)_ = 19.66, p<0.001), together with a significant “stimulus type”×“group” interaction (F_(1,28)_ = 4.51, p = 0.043). Further analyses revealed that the target-elicited P300 latency was longer than the distracter P300 latency in both groups (t_14_ = 9.73, and t_14_ = 5.91 for PD patients and HCs, respectively, p<0.001). For target stimuli, the P300 latency was longer in PD patients than in HCs (t_28_ = 3.19, p = 0.004). No other main effects or interactions were observed.

### Localization of P300 cortical generators with the swLORETA method

**Figure 4:**
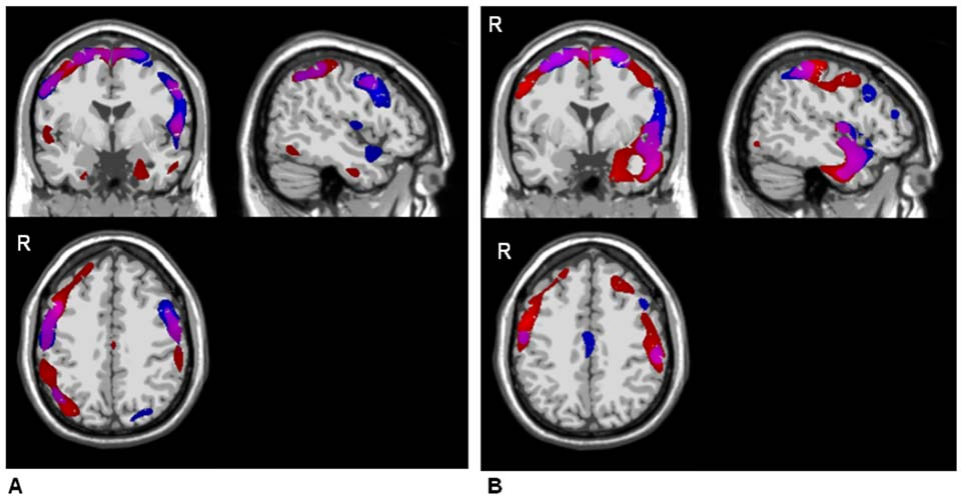
Colour-coded statistical maps of the P300 components grey matter current density. Each map was associated with a colour system (blue: healthy controls; red: PD patients). The colours were superimposed and areas of overlap (cortical regions showing significant generators for both groups) are displayed in the appropriate colour mixture (i.e. violet); (**SPM5® one sample t-tests**, swLORETA method p<0.0005). **4A:** distracter- elicited P300 components; **4B:** target-elicited P300 components.

**Figure 5:**
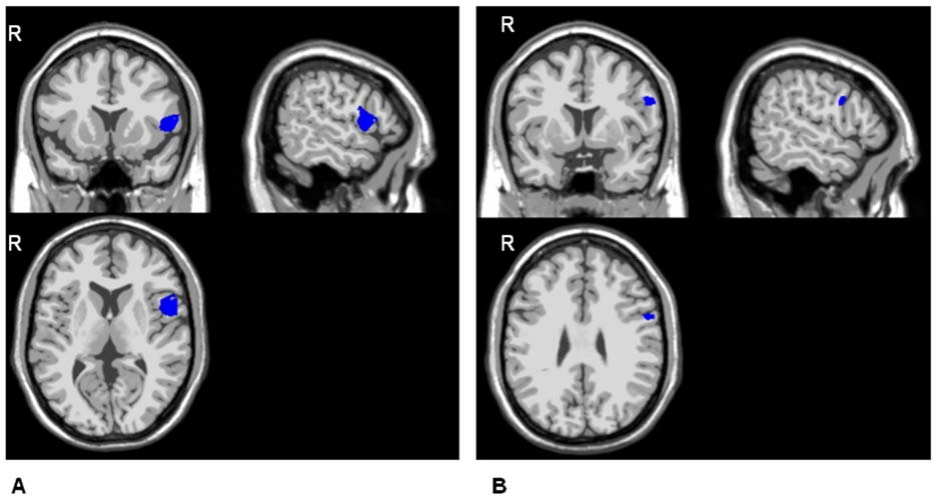
Between-group-statistical maps of the grey matter current density for the P300 components. Healthy controls versus PD patients (from SPM5® two sample t-tests). 5A: pooled distracter- and target- elicited P300 components. 5B: the distracter-elicited P300 component only (swLORETA method, p<0.005).

The swLORETA t-test maps are shown in Figures 4 and 5.

#### One sample t-tests

In HCs, both distracter- and target-elicited P300 generators appeared to be quite focused within frontoparietal areas, as shown in Figure 4. Distracter-elicited P300 generators were found mainly bilaterally in the middle, frontal, precentral, inferior and superior parietal lobules and the postcentral gyri and in the right superior and inferior frontal gyri. Some distracter-elicited P300 generators were also found in the bilateral lateral occipital gyri. The target-elicited P300 was generated in the bilateral superior frontal, precentral, postcentral gyri and superior parietal lobules, the left middle, frontal, superior and middle temporal gyri and inferior parietal lobule and, to a lesser extent, the right posterior cingulum and left insula.

In the PD patients, the distracter-elicited P300 generators were more widespread than in HCs, and primarily involved frontoparietal areas (the right middle and bilateral superior frontal and precentral gyri, the bilateral inferior parietal lobules and postcentral gyri and the right superior parietal lobule). However, temporal and occipital source locations (the bilateral parahippocampal gyri, the right lateral temporo-occipital and superior temporal and the left inferior temporal gyri) were also found. The target-elicited P300 component had the same frontal generators as the distracter-elicited P300 but differed slightly in terms of the parietal lobe generators (left inferior parietal, bilateral superior parietal lobules and the bilateral postcentral gyri). Generators were also identified in the temporal and occipital areas, with left predominance (the left parahippocampal, superior, middle and anterior temporal gyri, the lateral temporo-occipital gyri, the left insula, bilateral latero-occipital areas and the right lingual gyrus).

#### Two sample t-tests

As shown in Figure 5a, application of a two-sample t-test with an HC-PD patient contrast to all P300 components revealed fewer generators in the left inferior frontal gyrus in PD patients than in HCs.

Figure 5b shows the same significant, inter-group difference in this gyrus when the contrast is applied to the distracter-elicited P300 only.

No significant group effect was found in a target-elicited P300 generator analysis.

The PD-HC contrast did not reveal any significant differences.

## Discussion

The primary objective of the present study was to use swLORETA to determine how basal ganglia can modulate the cortical areas involved in the generation of distracter- and target-elicited P300 components when subjects perform an attention task that involves both control of selection and implementation of selection [3] (as is the case in a three-stimulus oddball paradigm). Parkinson's disease patients were compared with matched HCs, in order to specify the role of the associative frontostriatal loops in attention. Our results confirmed the involvement of frontoparietal networks in both distracter and target detection in PD patients and HCs, as already evidenced in young healthy subjects [6]. One of our most important findings relates to the significantly lower number of distracter-related P300 generators in the left inferior frontal gyrus (namely in its external and superior parts) in PD patients relative to HCs. There were no group differences for the target-elicited P300 sources. These results are in good agreement with the behavioral data, since PD patients had a higher commission rate for distracters (as already reported for other tasks) [80]–[83]. Our swLORETA results are also consistent with the scalp P300 amplitude distribution, showing a lack of frontal predominance in our PD group (regardless of the type of stimulus). Indeed, our HCs displayed frontocentral predominance for scalp P300, as already been shown in other studies of elderly HCs [84]–[87]. This finding contrasts with the situation usually observed in young subjects (particularly for target P300) [5], [6], [10]. According to Fabiani et al. [87], this age-related change may be related to a continuous engagement of working-memory processes - possibly because memory templates decay faster in old age or because greater susceptibility to distracters in the elderly may lead to an increased workload and thus greater recruitment of the frontal areas. This type of frontal recruitment may be less active in PD than in HCs, due to dysfunction of the frontostriatal circuits. Our swLORETA results also show that at the cortical level, the reduction in frontal generators preferentially concerns the distracter P300.

The areas in which distracter-elicited P300 generators were found in HCs but not PD patients correspond to the DLPF cortex, which is part of the DFP network and is involved in the executive frontostriatal loop [28]. Our data suggest that the attention impairment seen in PD patients is more related to DLPF cortex dysfunction than VLPF cortex dysfunction. This is in good agreement with Owen et al.'s suggestion that the function of the VLPF cortex is relatively intact in PD [88] and with the results of task-switching studies [89]. Our results are also in good agreement with neuroimaging studies that show DLPF dysfunction when PD patients are performing cognitive tasks. Indeed, several PET studies have revealed changes in blood flow in the DLPF and hypometabolism in the basal ganglia (the putamen, caudate nucleus or internal globus pallidus) [90]–[94]. Likewise, correlations between frontal [18F]-fluorodopa uptake and performance in working memory and verbal fluency tests have been evidenced in PD [95].

The prefrontal dysfunction in PD is considered to result from the disruption of the basal ganglia outflow resulting from dopamine depletion. In turn, this interrupts frontostriatal circuits [28] and may induce hypometabolism in frontal regions (including the DLPF cortex, the supplementary motor area, and the anterior cingulate gyrus) [96]. In particular, functional alteration of the DLPF cortex is likely in PD; the fact that dopamine modulates DLPF blood flow during planning tasks (in the absence of any change in basal ganglion blood flow) [97] suggests direct receptivity of the DLPF cortex to dopamine [97].

In our PD patients, only distracter-P300 generators were lacking in the DLPF cortex; there were no differences in target-P300 sources in this area (compared with HCs). This observation suggests that the DLPF dysfunction seen in attention tasks with basal ganglia impairment mainly involves distracter processing, whereas target detection does not appear to be markedly affected. Our PD patients were just as able to detect target stimuli as HCs were. Hence, the attention disorder in PD may be mostly related to the impaired inhibition of irrelevant stimuli. Indeed, by using a set-switching paradigm, Cools et al. [98] evidenced impaired cognitive control in PD and suggested that the patients' attention was captured more easily by salient information.

One remaining question relates to the origin of the specific DFP dysfunction revealed by tasks involving distracters. Is the dysfunction directly related to the primary basal ganglion impairment or does it depend on connections between the basal ganglia, the DLPF cortex and another structure (for example the anterior cingulate cortex (ACC))? We did not evidence P300 generators in the ACC, even though involvement in inhibition, response selection and conflict monitoring has been reported in previous studies [27], [99], [100]. The ACC has also been frequently related to the generation of an earlier cognitive ERP (the anterior N200) - mainly in go-no go tasks - [101] and is known to interact with the DLPF cortex [99], [102]. In particular, a conflict signal from the ACC could help recruit additional cognitive control functions carried out by the DLPF cortex [99]. It can reasonably be supposed that the ACC plays a role in inhibition in an earlier time window than the DLPF cortex does. Further investigation of the dynamic interaction between these two structures is thus merited.

We are aware that the present study has a number of limitations. Firstly, we deliberately chose to use an oddball paradigm - the most common paradigm for studying P300 and the mechanisms of attention. This choice is open to criticism, since other paradigms (such as two-stimulus go/no-go tasks with a high target probability and no distracters [101] are probably more appropriate for evaluating inhibition. However, the latter do not allow investigation of specific distracter and target processing and thus would not have matched our primary objective. Paradigms studying selective visuospatial attention might also be more appropriate for studying selective attention [103] (cf. studies on selective auditory attention in PD [56], [104], [105]) but could also introduce confounding factors, since visuospatial perception is impaired in PD [106], [107]. Furthermore, our paradigm could have been improved by simultaneously manipulating the difficulty of target vs. standard discrimination and modifying the distracter, as shown in previous studies [23], [108]. This would have allowed (i) better task performance and thus (possibly) the generation of more intense P300 and (ii) facilitated investigation of the relationships between stimulus-driven and task-related processes. However, our P300 subcomponents were clearly identifiable; given our focus on generator location, we wanted to use the same paradigm as in Bledowski et al. [66] and one of our previous studies [6].

Secondly, most of the patients had a mild form of PD, with very mild cognitive disorders, as evidenced by the extensive cognitive assessment (see Supporting Information S1). Nevertheless, the PD patients' impairment in distracter processing was revealed by a higher commission rate in the oddball task. This impairment was related to a difference (relative to HCs) in distracter-elicited P300 source locations. These findings suggest that (i) the function of the corticostriatal associative loop is altered in early-stage PD and (ii) cortical attention networks are modulated by the basal ganglia. Even though recruitment of PD patients with more severe cognitive impairments may have better highlighted differences with respect to HCs, it would also have raised several issues. For example, a lack of specificity in the patients' cognitive disorders would interfere with the results. Later-stage PD would also have prevented good task performance and thus decreased the robustness of the ERP analysis.

Thirdly, the male-to-female gender ratio in this study was 2.0, which is quite unbalanced and may be a possible source of bias. Nevertheless, this ratio is very similar to that reported in epidemiologic studies of PD patients [109], [110] and confirms the representativeness of our sample.

Fourthly, a significant group difference was observed for the score at the MADRS. PD patients had a slightly higher score than the HCs but it was well below the threshold of clinical significance and none met the diagnosis criteria for depression. This is usual in PD since some items of the MADRS can overlap with PD symptoms (sleep difficulties, anxiety…). It is very unlikely that this may have influenced our results.

Lastly, all patients in the present study were assessed on-drug; this may represent a confounding factor, since dopamine replacement therapy could either minimize differences between PD patients and HCs in terms of performance and P300 features or modify the function of the corticosubcortical networks. Nevertheless, the motor symptoms and lack of motivation in off-drug patients would have jeopardized task performance and compromised our ERP analysis.

In conclusion, we have shown that PD patients display fewer distracter-P300 generators in the DLPF cortex during a three-stimulus oddball paradigm. This finding suggests dysfunction of the dorsal frontoparietal attentional network when the basal ganglia are impaired and provides evidence for the modulation of cortical frontoparietal networks by the basal ganglia. Our data also indicate that the inhibition deficit in PD is probably related to less intense recruitment of the inferior frontal cortex following basal ganglia impairment. These results encourage the use of other electrophysiological methods (such as the analysis of rhythm oscillations during distracter and target processing) to further investigate the relationships and degree of coordination between these networks.
